# Analysis of the 10q23 chromosomal region and the *PTEN* gene in human sporadic breast carcinoma

**DOI:** 10.1038/sj.bjc.6690115

**Published:** 1999-02

**Authors:** H E Feilotter, V Coulon, J L McVeigh, A H Boag, F Dorion-Bonnet, B Duboué, W C W Latham1, C Eng, L M Mulligan, M Longy

**Affiliations:** Department of Pathology, Queen's University, Kingston, Ontario K7L 3N6, Canada; Molecular Oncology Laboratory, Institut Bergonié, 180, rue de Saint-Genès, Bordeaux, 33076, France; Department of Adult Oncology and the Charles A Dana Human Cancer Genetics Unit, Dana-Farber Cancer Institute, Department of Medicine, Harvard Medical School, Boston, MA, 02115, USA

**Keywords:** breast carcinoma, chromosome 10q, Cowden disease, *PTEN*

## Abstract

We examined a panel of sporadic breast carcinomas for loss of heterozygosity (LOH) in a 10-cM interval on chromosome 10 known to encompass the *PTEN* gene. We detected allele loss in 27 of 70 breast tumour DNAs. Fifteen of these showed loss limited to a subregion of the area studied. The most commonly deleted region was flanked by * D10S215* and *D10S541* and encompasses the *PTEN* locus. We used a combination of denaturing gradient gel electrophoresis and single-strand conformation polymorphism analyses to investigate the presence of *PTEN* mutations in tumours with LOH in this region. We did not detect mutations of *PTEN* in any of these tumours. Our data show that, in sporadic breast carcinoma, loss of heterozygosity of the *PTEN* locus is frequent, but mutation of *PTEN* is not. These results are consistent with loss of another unidentified tumour suppressor in this region in sporadic breast carcinoma. © 1999 Cancer Research Campaign


*British Journal of Cancer* (1999) **79**(5/6), 718–723
© 1999 Cancer Research Campaign
Article no. bjoc.1998.0115

### Analysis of the 10q23 chromosomal region and the *PTEN* gene in human sporadic breast carcinoma

**HE Feilotter[1]*, V Coulon[2]*, JL McVeigh[1]*, AH Boag[1], F Dorion-Bonnet[2], B Duboué[2], WCW Latham[1], C Eng[3], LM Mulligan[1] and M Longy[2]**

[1]Department of Pathology, Queen's University, Kingston, Ontario K7L 3N6, Canada; [2]Molecular Oncology Laboratory, Institut Bergonié, 180, rue de Saint-Genès, 33076 Bordeaux, France; [3]Department of Adult Oncology and the Charles A Dana Human Cancer Genetics Unit, Dana-Farber Cancer Institute, Department of Medicine, Harvard Medical School, 1 Jimmy Fund Way, Boston, MA 02115, USA

**Summary** We examined a panel of sporadic breast carcinomas for loss of heterozygosity (LOH) in a 10-cM interval on chromosome 10 known to encompass the *PTEN* gene. We detected allele loss in 27 of 70 breast tumour DNAs. Fifteen of these showed loss limited to a subregion of the area studied. The most commonly deleted region was flanked by *D10S215* and *D10S541* and encompasses the *PTEN* locus. We used a combination of denaturing gradient gel electrophoresis and single-strand conformation polymorphism analyses to investigate the presence of *PTEN* mutations in tumours with LOH in this region. We did not detect mutations of *PTEN* in any of these tumours. Our data show that, in sporadic breast carcinoma, loss of heterozygosity of the *PTEN* locus is frequent, but mutation of *PTEN* is not. These results are consistent with loss of another unidentified tumour suppressor in this region in sporadic breast carcinoma.

**Keywords:** breast carcinoma; chromosome 10q; Cowden disease; *PTEN*

Breast cancer affects one in nine women in their lifetime (Kelsey, 1979). In more than 90% of cases, the disease arises sporadically, with no previous associated family history. Several genes (e.g. *BRCA1*, *BRCA2*) have been shown to have a role in susceptibility to inherited breast cancer. Because of this association, such genes have been extensively investigated in sporadic breast tumours (reviewed in Smith and Ponder, 1993; Stratton, 1996); however, the genetic events occurring in the majority of these tumours still remain to be identified. Additional candidate genes for involvement in sporadic breast disease are those implicated in cancer syndromes associated with an increased risk of breast cancer. One such syndrome is Cowden disease (CD), or multiple hamartoma syndrome, an autosomal dominantly inherited cancer syndrome characterized by oral and cutaneous lesions and an increased risk of follicular thyroid tumours and breast carcinomas (Brownstein et al, 1978; Starink et al, 1986). The incidence of breast cancer among women with CD is estimated to be 30–50% (Starink et al, 1986).

The gene for CD was initially mapped by linkage analysis to 10q22–23, within a 5-cM interval flanked by *D10S215* and *D10S564* (Nelen et al, 1996). Loss of heterozygosity (LOH) of this interval was also observed in some tumours from CD patients (Marsh et al, 1997), suggesting the CD gene was a tumour suppressor. In addition to those tumours associated with CD, other human cancers have been shown to have losses of this region of chromosome 10 (Moschonas et al, 1996). A candidate tumour-suppressor gene for glioblastoma multiforme has been mapped to 10q24–qter (Karlbom et al, 1993; Pershouse et al, 1993; Rasheed et al, 1995; Albarosa et al, 1996), whereas a region in 10q23–q26 is frequently deleted in endometrial cancers (Simon et al, 1990; Peiffer et al, 1995). Common deleted regions have also been noted in renal cell carcinoma at 10q21–q22 (Morita et al, 1991), at 10q22–qter in malignant melanoma (Parmiter et al, 1988; Herbst et al, 1994), at 10q22.3–q23.1 in muscle-invasive bladder cancers (Kagan et al, 1998) and at 10q23–q24 in prostate cancer (Gray et al, 1996; Ittmann, 1996; Trybus et al, 1996; Feilotter et al, 1998).

Germline mutations of the *PTEN*/*MMAC1* tumour-suppressor gene (Li et al, 1997; Steck et al, 1997), which lies within the 10q22–23 critical interval, have been identified in the majority of CD families (Liaw et al, 1997; Lynch et al, 1997; Nelen et al, 1997; Tsou et al, 1997; Marsh et al, 1998*a*). However, mutations are rare or absent in breast cancer families without other typical CD stigmata (Tsou et al, 1997; Chen et al, 1998; FitzGerald et al, 1998). Somatic mutations of this gene have been found at varying frequencies in some of the tumour types described above. *PTEN* mutations are frequent in endometrial carcinoma (Kong et al, 1997; Risinger et al, 1997; Tashiro et al, 1997), malignant melanoma (Guldberg et al, 1997), high-grade gliomas (Rasheed et al, 1997; Wang et al, 1997; Bostrom et al, 1998) and metastatic prostate cancer (Cairns et al, 1997; Suzuki et al, 1998). However, mutations are rare in unselected prostate carcinoma (Feilotter et al, 1998), meningioma (Bostrom et al, 1998), low-grade gliomas (Rasheed et al, 1997) and unselected breast carcinoma (Rhei et al, 1997). Thus, it appears that the *PTEN* tumour suppressor may play a role in a subset of the advanced human cancers noted above, but not in all of these neoplasms. It is still not clear whether other tumour-suppressor genes which contribute to multiple tumour types lie close to *PTEN* on 10q23. In this study, we have investigated LOH in the 10q22–23 region in a series of primary breast carcinomas. We have further analysed those tumours with LOH in

*Received 5 August 1997*
*Revised 13 May 1998*
*Accepted 7 July 1998*

*Correspondence to:* LM Mulligan, Department of Paediatrics, 20 Barrie Street, Queen's University, Kingston, Ontario K7L 3N6, Canada

*These authors contributed equally to this work.

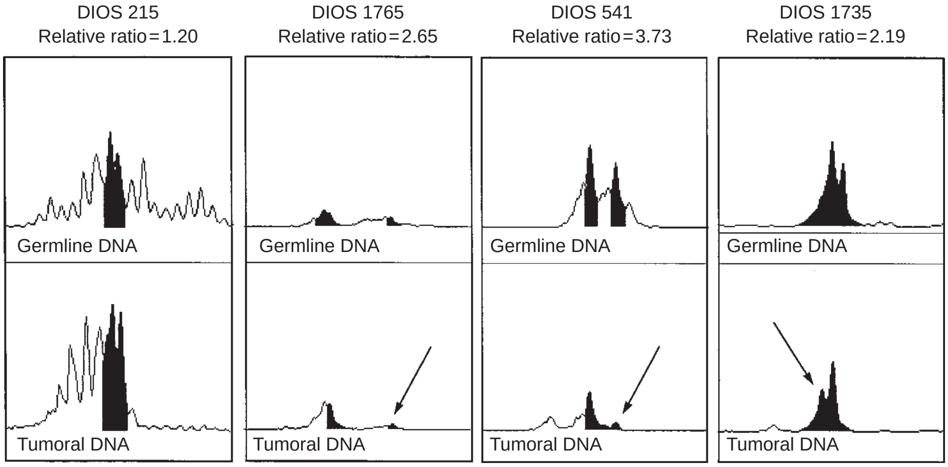

**Figure 1** Loss of heterozygosity analysis in tumour 22 for four informative markers. For each marker, the alleles present in normal (top) and tumour (bottom) tissue are shown, with the ratio of normal/tumour allele peak heights given above. Ratios equal to or greater than 1.6 were scored as allele loss. A ratio of less than 1.5 was scored as no loss. Thus, the figure depicts allele loss at *D10S1765*, *D10S541* and *D10S1735* and no loss at *D10S215*. The arrows indicate the deleted alleles in the tumour samples this critical region for mutations of the *PTEN* gene to clarify the role of *PTEN*, or closely linked tumour-suppressor genes, in this disease.

### MATERIALS AND METHODS

### Samples

We used a panel of 70 unselected sporadic primary breast cancers and matched normal tissue from two series for LOH on chromosome 10q, with particular attention to a 10-cM interval at 10q23 which spans the *PTEN* locus. Sixty-eight of the 70 samples were infiltrating ductal carcinomas, whereas the remaining two were infiltrating lobular carcinomas. The selected cases did not have a history of familial breast cancer or other cancer-related syndromes. Samples collected at the Institut Bergonié (cases 3–36) were isolated from fresh surgical material and snap-frozen in liquid nitrogen before DNA extraction. Peripheral blood leucocytes were obtained as a source of matched constitutional DNA. Genomic DNA purification from blood and snap-frozen tissue samples was according to published protocols (Dorion-Bonnet et al, 1995). DNA from samples collected at Kingston General Hospital (cases 37–72) was extracted from formalin-fixed paraffin-embedded archival material according to published protocols (Wright and Manos, 1990). Before each DNA extraction, tumour tissues were microdissected from contaminating normal cells using a haematoxylin phloxine saffron-stained section for reference. Control blood DNAs from a panel of 60 unrelated individuals were extracted using established protocols and have been previously described (Feilotter et al, 1998).

### LOH analyses

Seven microsatellite markers, known to map to the 10q23 interval, were analysed. The order of these markers from centromere to telomere was *D10S579*, *D10S215*, *D10S1765*, *D10S541*, *D10S1735*, *D10S1739*, *D10S564* based on published maps (Gyapay et al, 1994; Chumakov et al, 1995; Moschonas et al, 1996; Nelen et al, 1996; Gray et al, 1997; Marsh et al, 1997) and data from the Center for Genome Research at the Whitehead Institute for Biomedical Science (www-genome.wi.mit.edu). Primer sequences for markers were obtained from the Genome Data Base. Polymerase chain reactions (PCR) were carried out according to published protocols using either fluorescently or radiolabelled primers. For fluorescent products, allele loss was scored when the ratio of peak heights of the tumour to the leucocyte alleles, as determined by Genescan 672 software (Applied Biosystems), exceeded 1.6 (Figure 1). This cut-off value represents a conservative scoring of allele loss, and is consistent with that used in other studies of LOH (Gray et al, 1996; Trybus et al, 1996; Marsh et al, 1997). Samples with a ratio of peak heights between 1.5 and 1.6 were considered ambiguous and were not scored. For radiolabelled products (samples 37–72), allele loss was scored by eye by two independent observers.

### Single-strand conformation polymorphism (SSCP) analysis

SSCP analysis of *PTEN* was performed using previously described PCR conditions and intronic primers flanking each of the gene's nine exons (Feilotter et al, 1998). Aliquots of the labelled PCR products were electrophoresed on non-denaturing acrylamide/glycerol gels at 14°C for 16 h using a variety of polyacrylamide and glycerol concentrations, ranging from 6% to 8% acrylamide and from 0% to 10% glycerol. Gels were dried and exposed to film.

### Denaturing gradient gel electrophoresis (DGGE) analysis

DGGE was performed according to the methods described by Stoppa-Lyonnet et al (1997). DGGE electrophoretic conditions (optimized using Meltmap and SQHTX programs) and primer sequences have been previously described (Laugé et al, 1998).

### Sequencing analysis

Electrophoretic variants predicted by SSCP or DGGE were confirmed by direct sequencing. PCR products were amplified

© Cancer Research Campaign 1999 

| Locus | 71 | 66 | 10 | 46 | 58 | 4 | 56 | 53 | 27 | 50 | 55 | 65 | 59 | 21 | 40 | 41 | 6 | 13 | 14 | 5 | 52 | 22 | 11 | 17 | 68 | 39 | 12 |
|---|---|---|---|---|---|---|---|---|---|---|---|---|---|---|---|---|---|---|---|---|---|---|---|---|---|---|---|
| D10S579 | na | na | – | – | ● | – | – | ● | – | na | na | – | ● | ● | – | na | – | ○ | – | – | na | ● | – | ● | ○ | ○ | ○ |
| D10S215 | ● | ● | – | – | – | – | – | – | – | – | na | – | na | – | ● | – | – | – | ○ | – | – | ○ | ○ | na | – | – | ● |
| D10S1765 | na | ● | na | ● | ● | na | ● | ● | – | ● | na | na | ● | ● | ● | ● | ● | ● | ● | ● | ● | ● | ● | ○ | ○ | ○ | ○ |
| D10S541 | ● | – | ● | ● | ● |  | ● | ● | ● | – | – |  | – | – | na | na | – | ○ | ○ | ○ | ○ | ● | – | ○ | ○ | ○ | – |
| D10S1735 | – | – | – | – | – | ● | – | – | ● | – | ● | ● | ○ | ○ | – | – | – | ○ | ○ | – | – | ● | – | ○ | ● | – | ○ |
| D10S1739 | – | – | – | na | – | ● | ● | ● | ● | – | ● | – | ○ | – | ○ | ○ | ○ | ○ | ○ | ○ | – | – | – | ○ | – | – | – |
| D10S564 | ● | – | – | – | – | – | ● | na | ● | ● | na | ● | na | ○ | ○ | ○ | ○ | ○ | ○ | – | – | – | – | – | – | ● | ○ |

| Size (mm) | 15 | 20 | 10 | 30 | 17 | 30 | 35 | 19 | 17 | 30 | 30 | 35 | 20 | 13 | 15 | 10 | 12 | 20 | 15 | 23 | 20 | 33 | 25 | 20 | 15 | 40 | 20 |
|---|---|---|---|---|---|---|---|---|---|---|---|---|---|---|---|---|---|---|---|---|---|---|---|---|---|---|---|
| Histological grade | P | P | P | P | P | MW | M | M | MW | S | M | P | P | MW | M | W | MW | MW | P | MW | M | MW | P | MW | M | P | MW |
| Lymph nodes No. positive | 0 | 0 | 3 | 1 | 0 | 1 | 0 | 0 | 0 | 0 | 0 | 0 | 2 | 1 | 1 | 0 | 0 | 2 | 1 | 0 | 0 | 3 | 0 | 4 | 0 | 0 | 3 |
| No. examined | 14 | 13 | 15 | 7 | 20 | 23 | 14 | 10 | 8 | 5 | 9 | 9 | 26 | 14 | 8 | 8 | 14 | 9 | 16 | 22 | 8 | 21 | 13 | 13 | 17 | 2 | 8 |

**Figure 2** Regions of allele loss for the 27 tumours showing LOH. Open circles = no LOH; filled circles = LOH, – = non-informative; na = not available. The shading represents the maximum possible interval of loss for each tumour. Grade for each tumour is given as W, well differentiated; M, moderately differentiated; MW, moderately/well differentiated; P, poorly differentiated, and S, special type, not graded (medullary carcinoma). Lymph node involvement is indicated as number of axillary lymph nodes with metastatic cancer over the total number of nodes examined essentially as described above (Feilotter et al, 1998; Longy et al, 1998) except without the addition of isotope. Products were purified using the Wizard PCR Preps DNA purification system (Promega) and sequenced using the Thermosequenase Sequencing Kit (Amersham-Life Science) or the ABI Prism Dye Terminator Sequencing Ready Reaction Kit (Perkin Elmer) according to the manufacturer's instructions.

### RESULTS

Of the seventy pairs of matched breast carcinoma and germline genomic DNAs examined, all were informative (heterozygous) for at least one of the markers analysed. In total, 27 tumours (39%) showed LOH at one or more marker loci. Twelve of these (71, 66, 10, 46, 58, 4, 56, 53, 27, 50, 55 and 65) exhibited allele loss for all informative markers over the entire 10-cM region examined (Figure 2). Thus, LOH in these tumours spanned extensive regions of 10q and was not useful for further defining the critical deleted interval or intervals. In the remaining 15 cases, LOH was detected at only a subset of the informative loci within the 10q23 region.

The most frequent allele loss was at *D10S1765*, although loss was common between *D10S215* and *D10S541*. Thirteen out of the 15 tumours which showed loss at only a subset of loci had LOH within this interval (59, 21, 40, 41, 6, 13, 14, 5, 52, 22, 11, 17 and 12) (Figure 2). This commonly deleted interval spans the *PTEN* locus.

In addition to the interval described above, a distinct non-overlapping region of allelic loss was seen in two samples (Figure 2). Tumours 68 and 39 showed allele loss only for markers telomeric of *D10S541*. Further, tumour 68 showed LOH at the more distal locus *D10S574* (data not shown), suggesting that the deleted interval spanned markers well telomeric of *D10S564* (Figure 2).

### *PTEN* mutation analyses

To investigate whether *PTEN* was the target of allele loss in tumours where LOH was detected, we examined all nine exons of the *PTEN* gene in each of the 27 tumours which showed allele loss at 10q23 loci. For each tumour sample and control, *PTEN* exons were amplified using intronic primers. PCR products with altered mobility (SSCP) or denaturation profiles (DGGE), suggesting the presence of mutations, were directly sequenced. SSCP and DGGE variants were detected with PCR products corresponding to exons 2, 4 and 6. Sequencing of these variants confirmed that these products represented previously reported polymorphic alleles present in the normal population (Rhei et al, 1997; Feilotter et al, 1998). We did not detect mutations of the *PTEN* gene in any of the 27 breast carcinomas examined in this study. We conclude that mutations of *PTEN* are not common in breast cancer.

### DISCUSSION

The *PTEN*/*MMAC1* gene lies between *D10S1765* and *D10S541* (Gray et al, 1996) within the major region of loss detected in this study. The gene encodes a molecule with homology to two cytoskeletal proteins, tensin and auxilin (Li et al, 1997; Steck et al, 1997). PTEN has demonstrated dual specificity phosphatase activity (Li and Sun, 1997; Myers et al, 1997), which suggests a role in both tyrosine and serine/threonine phosphatase-mediated signal transduction.

Although frequent mutations of *PTEN* in a variety of tumour types was predicted from early studies of tumour cell lines, this has not been generally confirmed. Analyses of *PTEN* in primary tumours have identified mutations in approximately one-third of high-grade gliomas (Rasheed et al, 1997; Wang et al, 1997; Bostrom et al, 1998) and about 50% of endometrial tumours (Kong et al, 1997; Risinger et al, 1997; Tashiro et al, 1997). However, mutations are common only in metastatic prostate

*© Cancer Research Campaign 1999*

carcinoma (Cairns et al, 1997; Feilotter et al, 1998; Suzuki et al, 1998) and malignant melanoma (Guldberg et al, 1997). In recent studies, it has been shown that germline *PTEN* mutations are rare or absent in cases of familial breast cancer or early onset breast cancer without *BRCA1* mutations (Tsou et al, 1997; Chen et al, 1998; FitzGerald et al, 1998). Li et al (1997) identified mutations leading to truncation of the PTEN protein in 2 out of 20 (10%) breast tumour cell lines examined. Studies in unselected series of primary breast tumours have not identified high frequencies of *PTEN* mutations in sporadic tumours (Rhei et al, 1997). In this study, we examined a selected population of tumours already shown to have LOH for the *PTEN* region, which might be predicted to have a higher frequency of *PTEN* mutations. Interestingly, our data are very similar to results from Rhei et al (1997) who found only one *PTEN* mutation in 53 truly sporadic tumours. The low frequency of *PTEN* mutations in breast cancer cell lines and primary tumours, even in studies such as ours in which samples were preselected for LOH in the *PTEN* region, raises the possibility that another tumour-suppressor gene with a role in sporadic breast cancers may lie within this interval on 10q23.

Genome-wide allelotyping studies of sporadic breast cancers have not shown LOH above background levels on chromosome 10 (Sato et al, 1990; Devilee and Cornelisse, 1994; Bieché and Lidereau, 1995; Fujii et al, 1996; Kerangueven et al, 1996). However, these studies did not focus on the 10q23 region. In this study, we saw LOH for one or more loci in 10q23 in 27 out of 70 sporadic breast carcinomas. In >90% of these cases, deletion included the *PTEN* locus. Singh et al (1998) reported a similar frequency of LOH (41%) for this interval in sporadic breast tumours whereas Li et al (1997) saw LOH of *PTEN* in approximately 50% of primary tumours examined. Our data and those of Singh et al (1998) suggest that the most common interval of loss spans approximately 1 Mb of DNA which, although including the *PTEN* locus, could also include one or more further genes. As yet, additional loci within this interval, which might act as tumour-suppressor genes in sporadic breast cancer, have not been identified. Clearly, the existence of such candidate genes, which are lost in the samples studied here, remains to be determined.

Analysis of LOH in a variety of tumours has suggested that multiple discrete regions of chromosome 10 loss may occur in a single tumour or tumour type (Parmiter et al, 1988; Karlbom et al, 1993; Herbst et al, 1994; Peiffer et al, 1995; Albarosa et al, 1996; Ittmann, 1996; Trybus et al, 1996; Zedenius et al, 1996; Marsh et al 1998*b*). The precise physical localization of such deleted regions with respect to one another and the number of tumour-suppressor genes they represent has been difficult to determine, in part because of the variety of markers used in the different studies. Our identification of a second distinct deleted interval telomeric of the *D10S215*/*D10S541* interval is consistent with the suggestion that other genes on 10q23, outside of the *PTEN* interval, may contribute to some sporadic breast cancers. LOH analyses of a larger population of sporadic breast tumours may help to clarify this issue. The loss of multiple distinct regions on chromosome 10 may be analogous to the situation on chromosome 17 in sporadic breast tumours, in which at least five distinct deleted regions have been recognized (Nagai et al, 1994, 1995). In the case of chromosome 17, the pattern of LOH is significantly correlated with some clinical parameters of the breast tumours (Bevilacqua et al, 1989; Nagai et al, 1995; Midulla et al, 1996). Our data do not suggest any association of chromosome 10 allele loss with tumour size, stage or lymph node involvement, regardless of the deleted interval (Figure 2), although our numbers are too small for subset analyses. Singh et al (1998) has suggested a strong correlation of LOH in 10q23 with poorly differentiated cancers, although again the numbers examined were small and *PTEN* mutation status was not determined. Further study is required to confirm whether losses can be correlated with these or other clinical parameters.

In summary, we have identified two discrete regions of LOH on 10q in sporadic breast carcinomas. Losses of the major region identified in this study were found in 39% of the cases studied and spanned the locus of the *PTEN* tumour suppressor. Mutations of *PTEN* were not identified in any of these cases, suggesting other candidate tumour-suppressor genes, important in breast cancer, may lie within the same region. A tumour population with allele losses in the 10q23 region, such as we have described here, will provide a valuable tool for determining the relative contribution of inactivation of the *PTEN* gene, or a neighbouring tumour-suppressor gene, to breast tumorigenesis.

### ACKNOWLEDGEMENTS

We thank Drs Bruce Elliot, Maria Nagai, Ramon Parsons and Paul Young for helpful discussions, Jean Luc Birac for preparation of figures and Noelle Robertson for assistance. This work was supported by the National Cancer Institute of Canada, the Kingston General Hospital Foundation, Le Comité National et les Comités Départementaux de la Correzé et de la Gironde de la Ligue Contre le Cancer, the Lawrence and Susan Marx Investigatorship in Human Cancer Genetics, the Concert for the Cure and the American Cancer Society (RPG-97-064-01-VM).

### REFERENCES

Albarosa R, Colombo BM, Roz L, Magnani I, Pollo B, Cirenei N, Giani C, Conti AMF, DiDonato S and Finocchiaro G (1996) Deletion mapping of gliomas suggests the presence of two small regions for candidate tumor-suppressor genes in a 17-cM interval on chromosome 10q. *Am J Hum Genet* **58**: 1260–1267

Bevilacqua G, Sobel ME, Liotta LA and Steeg PS (1989) Association of low *nm23* RNA levels in human primary infiltrating ductal breast carcinomas with lymph node involvement and other histopathological indicators of high metastatic potential. *Cancer Res* **49**: 5185–5190

Bieché I and Lidereau R (1995) Genetic alterations in breast cancer. *Genes Chrom Cancer* **14**: 227–251

Bostrom J, Cobbers JMJL, Wolter M, Tabatabai G, Weber RG, Lichter P, Collins VP and Reifenberger G (1998) Mutation of the PTEN (MMACI) tumor suppressor gene in a subset of glioblastomas but not in meningiomas with loss of chromosome arm 10q. *Cancer Res* **58**: 29–33

Brownstein MH, Wolf M and Bikowski JB (1978) Cowden's disease: a cutaneous marker of breast cancer. *Cancer* **41**: 2393–2398

Cairns P, Okami K, Halachmi S, Halachmi N, Esteller M, Herman JG, Jen J, Isaacs WB, Bova GS and Sidransky D (1997) Frequent inactivation of PTEN/MMAC1 in primary prostate cancer. *Cancer Res* **57**: 4997–5000

Chen J, Lindblom P and Lindblom A (1998) A study of the *PTEN*/*MMAC1* gene in 136 breast cancer families. *Hum Genet* **102**: 124–125

Chumakov IM, Rigault P, Le Gall I, Bellanne-Chantelot C, Billault A, Guillou S, Soularue P, Guasconi G, Poullier E, Gros I, Belova M, Sambucy J, Susini L, Gervy P, Glibert F, Beaufils S, Bui H, Massart C, De Tand M, Dukasz F, Lecoulant S, Ougen P, Perrot V, Saumier M, Soravito C, Bahouayila R, Cogen-Akenine A, Barillot E, Bertrand S, Codani J, Caterina D, Georges I, Lacroix B, Lucotte G, Sahbatou M, Schmit C, Sangouard M, Tubacher E, Dib C, Faure S, Fizames C, Gyapay G, Millasseau P, Nguyen S, Muselet D, Vignal A, Morissette J, Menninger J, Lieman J, Desai T, Banks A, Bray-Ward P, Ward D, Hudson T, Gerety S, Foote S, Stein L, Page DC, Lander ES, Weissenbach J, Le Paslier D and Cohen D (1995) A YAC contig map of the human genome. *Nature* **377**: 175–297

© Cancer Research Campaign 1999

Devilee P and Cornelisse CJ (1994) Somatic genetic changes in human breast cancer. *Biochim Biophys Acta* **1198**: 113–130
Dorion-Bonnet F, Mautalen S, Hostein I and Longy M (1995) Allelic imbalance study of 16q in human primary breast carcinomas using microsatellite markers. *Genes Chromosomes Cancer* **14**: 171–181
Feilotter HE, Nagai MA, Boag AH, Eng C and Mulligan LM (1998) Analysis of *PTEN* and the 10q23 region in primary prostate carcinomas. *Oncogene* **16**: 1743–1748
FitzGerald MG, Marsh DJ, Wahrer D, Bell D, Caron S, Shannon KE, Ishioka C, Isselbacher KJ, Garber JE, Eng C and Haber DA (1998) Germline mutations in *PTEN* are an infrequent cause of genetic predisposition to breast cancer. *Oncogene* **17**: 727–731
Fujii H, Szumel R, Marsh C, Zhou W and Gabrielson E (1996) Genetic progression, histological grade, and allelic loss in ductal carcinoma in situ of the breast. *Cancer Res* **56**: 5260–5265
Gray IC, Phillips SMA, Lee SJ, Neoptolemos JP, Weissenbach J and Spurr NK (1996) Loss of the chromosomal region 10q23–25 in prostate cancer. *Cancer Res* **55**: 4800–4803
Gray IC, Fallowfield J, Ford S, Nobile C, Volpi EV and Spurr NK (1997) An integrated physical and genetic map spanning chromosome band 10q24. *Genomics* **43**: 85–88
Guldberg P, thor Straten P, Birck A, Ahrenkiel V, Kirkin AF and Zeuthen J (1997) Disruption of the MMACI/PTEN gene by deletion or mutation is a frequent event in malignant melanoma. *Cancer Res* **57**: 3660–3663
Gyapay G, Morissette J, Vignal A, Dib C, Fizames C, Millasseau P, Marc S, Benardi G, Lathrop M and Weissenbach J (1994) The 1993–94 Genethon human genetic linkage map. *Nature Genet* **7**: 246–339
Herbst RA, Weiss J, Ehnis A, Cavenee WK and Arden KC (1994) Loss of heterozygosity for 10q22–10qter in malignant melanoma progression. *Cancer Res* **54**: 3111–3114
Ittmann M (1996) Allelic loss on chromosome 10 in prostate adenocarcinoma. *Cancer Res* **56**: 2143–2147
Kagan J, Liu J, Stein JD, Wagner SS, Babkowski R, Grossman BH and Katz RL (1998) Cluster of allele losses within a 2.5 cM region of chromosome 10 in high-grade invasive bladder cancer. *Oncogene* **16**: 909–913
Karlbom AE, James CD, Boethius J, Cavenee WK, Collins VP, Nordenskjöld M and Larsson C (1993) Loss of heterozygosity in malignant gliomas involves at least three distinct regions on chromosome 10. *Hum Genet* **92**: 169–174
Kelsey J (1979) A review of the epidemiology of human breast cancer. *Epidemiol Rev* **1**: 74–109
Kerangueven F, Eisinger F, Noguchi T, Allione F, Wargniez V, Eng C, Padberg G, Theillet C, Jacquenier J, Longy M, Sobol H and Birnbaum D (1996) Loss of heterozygosity in human breast carcinomas in the ataxia telangiectasia, Cowden disease and *BRCA1* gene regions. *Oncogene* **13**: 339–347
Kong D, Suzuki A, Zou TT, Sakurada A, Kemp LW, Wakatsuki S, Yokoyama T, Yamakawa H, Furukawa T, Sato M, Ohuchi N, Sato S, Yin J, Wang S, Abraham JM, Souza RF, Smolinski KN, Meltzer SJ and Horii A (1997) PTEN1 is frequently mutated in primary endometrial carcinomas. *Nature Genet* **17**: 143–144
Laugé A, Lefèbvre C, Laurent-Puig P, Gad S, Eng C, Longy M & Stoppa-Lyonnet D (1998) No evidence for germline *PTEN* mutations in families with breast and brain tumours. *Int J Cancer* (submitted)
Li D-M and Sun H (1997) TEP1, encoded by a candidate tumor suppressor locus is a novel protein tyrosine phosphatase regulated by transforming growth factor β. *Cancer Res* **57**: 2124–2129
Li J, Yen C, Liaw D, Podsypanina K, Bose S, Wang SI, Puc J, Miliaresis C, Rodgers L, McCombie R, Bigner SH, Giovanella BC, Ittmann M, Tycko B, Hibshoosh H, Wigler MH and Parsons R (1997) *PTEN*, a putative protein tyrosine phosphatase gene mutated in human brain, breast, and prostate cancer. *Science* **275**: 1943–1947
Liaw D, Marsh DJ, Li J, Dahia PLM, Wang SI, Zheng Z, Bose S, Call KM, Tsou HC, Peacocke M, Eng C and Parsons R (1997) Germline mutations of the *PTEN* gene in Cowden disease, an inherited breast and thyroid cancer syndrome. *Nature Genet* **16**: 64–67
Longy M, Coulon V, Duboué B, David A, Larrègue M, Eng C, Amati P, Kraimps J-L, Bottani A, Lacombe D and Bonneau D (1998) Mutations of PTEN in patients with Bannayan-Riley-Ruvalcaba phenotype. *J Med Genet* (in press)
Lynch ED, Ostermeyer EA, Lee MK, Arena JF, Ji H, Dann J, Swisshelm K, Suchard D, MacLeod PM, Kvinnsland S, Gjertsen BT, Heimdal K, Lubs H, Moller P and King M-C (1997) Inherited mutations in PTEN that are associated with breast cancer, Cowden disease, and juvenile polyposis. *Am J Hum Genet* **61**: 1254–1260
Marsh DJ, Zheng Z, Zedenius J, Kremer H, Padberg GW, Larsson C, Longy M and Eng C (1997). Differential loss of heterozygosity in the region of the Cowden locus within 10q22–23 in follicular thyroid adenomas and carcinomas. *Cancer Res* **57**: 500–503
Marsh DJ, Coulon V, Lunetta KL, Rocca-Serra P, Dahia PLM, Zheng Z, Liaw D, Caron S, Duboue B, Lin AY, Richardson A-L, Bonnetblanc J-M, Bressieux J-M, Cabarrot-Moreau A, Chompret A, Demange L, Eeles RA, Yahanda AM, Fearon ER, Fricker J-P, Gorlin RJ, Hodgson SV, Huson S, Lacombe D, LePrat F, Odent S, Toulouse C, Olopade OI, Sobol H, Tishler S, Woods CG, Robinson BG, Weber HC, Parsons R, Peacocke M, Longy M and Eng C (1998*a*) Mutation spectrum and genotype–phenotype analyses in Cowden disease and Bannayan–Zonana syndrome, two hamartoma syndromes with germline PTEN mutation. *Hum Mol Genet* **7**: 507–515
Marsh DJ, Dahia PLM, Coulon V, Zheng Z, Dorion-Bonnet F, Call KM, Little R, Lin AY, Eeles RA, Goldstein AM, Hodgson SV, Richardson A-L, Robinson BG, Weber HC, Longy M and Eng C (1998*b*) Allelic imbalance, including deletion of *PTEN*/*MMAC1*, at the Cowden disease locus on 10q22–23, in hamartomas from patients with Cowden syndrome and germline *PTEN* mutation. *Genes Chromosomes Cancer* **21**: 61–69
Midulla C, Deiorio P, Valli C, Felici A, Serpiesi DE, Marzetti L, Nofrani I and Vecchione A (1996) nm23 and p53 expression: correlation with DNA ploidy and other pragmatic parameters in breast cancer. *Oncol Rep* **3**: 957–961
Morita R, Saito S, Ishikawa J, Ogawa O, Yoshida O, Yamakawa K and Nakamura Y (1991) Common regions of deletion on chromosomes 5q, 6q and 10q in renal cell carcinoma. *Cancer Res* **51**: 5817–5820
Moschonas NK, Spurr NK and Mao J (1996) Report of the first international workshop on human chromosome 10 mapping 1995. *Cytogenet Cell Genet* **72**: 99–112
Myers MP, Stolarov JP, Eng C, Li J, Wang SI, Wigler MH, Parson R and Tonks NK (1997) PTEN, the tumor suppressor from human chromosome 10q23, is a dual-specificity phosphatase. *Proc Natl Acad Sci USA* **94**: 9052–9057
Nagai MA, Yamamoto L, Salaorni S, Pacheco MM, Brentani MM, Barbosa EM, Brentani RR, Mazoyer S, Smith SA, Ponder BAJ and Mulligan LM (1994) Detailed deletion mapping of chromosome segment 17q12–21 in sporadic breast tumours. *Genes Chromosomes Cancer* **11**: 58–62
Nagai MA, Medeiros AC, Brentani MM, Brentani RR, Marques LA, Mazoyer S and Mulligan LM (1995) Five distinct deleted regions on chromosome 17 defining different subsets of human primary breast tumors. *Oncology* **52**: 448–453
Nelen MR, Padberg GW, Peeters EAJ, Lin AY, van den Helm B, Frants RR, Coulon V, Goldstein AM, van Reen MMM, Easton DF, Eeles RA, Hodgson S, Mulvihill JJ, Murday VA, Tucker MA, Mariman ECM, Starink TM, Ponder BAJ, Ropers HH, Kremer H, Longy M and Eng C (1996) Localization of the gene for Cowden disease to chromosome 10q22–23. *Nature Genet* **13**: 114–116
Nelen MR, van Staveren WCG, Peeters EAJ, Hassel MB, Gorlin RJ, Hamm H, Lindboe CF, Fryns J-P, Sijmons RH, Woods DG, Mariman ECM, Padberg GW and Kremer H (1997) Germline mutations in the *PTEN*/*MMAC1* gene in patients with Cowden disease. *Hum Mol Genet* **6**: 1383–1387
Parmiter AH, Balaban G, Clark WHJ and Nowell PC (1988) Possible involvement of the chromosome region 10q24–26 in early stages of melanocytic neoplasia. *Cancer Genet Cytogenet* **30**: 313–317
Peiffer SL, Herzog TJ, Tribune DJ, Mutch DG, Gersell DJ and Goodfellow PJ (1995) Allelic loss of sequences from the long arm of chromosome 10 and replication errors in endometrial cancers. *Cancer Res* **55**: 1922–1926
Pershouse MA, Stubblefield E, Hadi A, Killary AM, Yung WKA and Steck PA (1993) Analysis of the functional role of chromosome 10 loss in human glioblastomas. *Cancer Res* **53**: 5043–5050
Rasheed BKA, McLendon RE, Friedman HS, Friedman AH, Fuchs HE, Bigner DD and Bigner SH (1995) Chromosome 10 deletion mapping in human gliomas: a common deletion region in 10q25. *Oncogene* **10**: 2243–2246
Rasheed BKA, Stenzel TT, McLendon RE, Parsons R, Friedman AH, Friedman HS, Bigner DD and Bigner SH (1997) PTEN gene mutations are seen in high-grade but not in low-grade gliomas. *Cancer Res* **57**: 4187–4190
Rhei E, Kang L, Bogomolniy F, Federici MG, Borgen PI and Boyd J (1997) Mutation analysis of the putative tumor suppressor gene PTEN/MMACI in primary breast carcinomas. *Cancer Res* **57**: 3657–3659
Risinger JI, Hayes AK, Berchuck A and Barrett JC (1997) *PTEN*/*MMAC1* mutations in endometrial cancers. *Cancer Res* **57**: 4736–4738
Sato T, Tanigami A, Yamakawa K, Akiyama F, Kasumi F, Sakamoto G and Nakamura Y (1990) Allelotype of breast cancer: cumulative allele losses promote tumor progression in primary breast cancer. *Cancer Res* **50**: 7184–7189
Simon D, Hener S, Satyaswaroop PG, Farber M and Noumoff JS (1990) Is chromosome 10 a primary chromosomal abnormality in endometrial adenocarcinomas? *Cancer Genet Cytogenet* **47**: 155–162
Singh B, Ittmann MM and Krolewski JJ (1998) Sporadic breast cancers exhibit loss of heterozygosity on chromosome segment 10q23 close to the Cowden disease locus. *Genes Chromosomes Cancer* **21**: 166–171

© Cancer Research Campaign 1999

Smith SA and Ponder BAJ (1993) Predisposing genes in breast and ovarian cancer: an overview. *Tumori* **79**: 291–296

Starink TM, van der Veen JPW, Arwert F, de Waal LP, de Lange GG, Gille JJP and Eriksson AW (1986) The Cowden syndrome: a clinical and genetic study in 21 patients. *Clin Genet* **29**: 222–233

Steck PA, Pershouse MA, Jasser SA, Yung WKA, Lin H, Ligon AH, Langford LA, Baumgard ML, Hattier T, Davis T, Frye C, Hu R, Swedlund B, Teng DHF and Tavtigian SV (1997) Identification of a candidate gene, *MMAC1*, at chromosome 10q23.3 that is mutated in multiple advanced tumours. *Nature Genet* **15**: 356–362

Stoppa-Lyonnet D, Laurent-Puig P, Essioux L, Pagès S, Ithier G, Ligot L, Fourquet A, Salmon RJ, Clough KB, Pouillart P, Bonaïti-Pellié C and Thomas G (1997) BRCA1 sequence variations in 160 individuals referred to a breast/ovarian family cancer clinic. *Am J Hum Genet* **60**: 1021–1030

Stratton MR (1996) Recent advances in understanding of genetic susceptibility to breast cancer. *Hum Mol Genet* **5**: 1515–1519

Suzuki H, Freije D, Nusskern DR, Okami K, Cairns P, Sidransky D, Isaacs WB and Bova GS (1998) Interfocal heterogeneity of PTEN/MMAC1 gene alterations in multiple metastatic prostate cancer tissues. *Cancer Res* **58**: 204–209

Tashiro H, Blazes MS, Wu, R, Cho KR, Bose S, Wang SI, Li J, Parsons R and Ellenson LH (1997) Mutations in PTEN are frequent in endometrial carcinoma but rare in other common gynecological malignancies. *Cancer Res* **57**: 3935–3940

Trybus TM, Burgess AC, Wojno KJ, Glover TW and Macoska JA (1996) Distinct areas of allelic loss on chromosomal regions 10p and 10q in human prostate cancer. *Cancer Res* **56**: 2263–2267

Tsou HC, Teng DH-F, Ping XL, Brancolini V, Davis T, Hu R, Xie XX, Gruener AC, Schrager CA, Christiano AM, Eng C, Steck P, Ott J, Tavtigian SV and Peacocke M (1997) The role of MMAC1 mutations in early-onset breast cancer: causative in association with Cowden syndrome and excluded in BRCA1-negative cases. *Am J Hum Genet* **61**: 1036–1043

Wang SI, Puc J, Li J, Bruce JN, Cairns P, Sidransky D and Parsons R (1997) Somatic mutations of PTEN in glioblastoma multiforme. *Cancer Res* **57**: 4183–4186

Wright DK and Manos MM (1990) Sample preparation from paraffin-embedded tissues. In *PCR Protocols*: *a Guide to Methods and Applications*, pp. 153–158. Academic Press: San Diego

Zedenius J, Wallin G, Svensson A, Bovée J, Höög A, Bäckahl M and Larsson C (1996) Deletions of the long arm of chromosome 10 in progression of follicular thyroid tumors. *Hum Genet* **97**: 299–303

*© Cancer Research Campaign 1999*
